# Clinical Insights into Risk Factors for Infantile Hemangioma and Propranolol Treatment Outcomes

**DOI:** 10.3390/diagnostics15141792

**Published:** 2025-07-16

**Authors:** Ioana Roșca, Raluca-Gabriela Miulescu, Alexandra-Maria Roman, Oana-Alexandra Peta, Alina Turenschi, Anca Miu, Aurelia Sosoi, Andreea Teodora Constantin, Leonard Năstase, Sânziana Miu, Alexandru Dinulescu, Elena Poenaru, Florica Șandru

**Affiliations:** 1 Faculty of Medicine, University of Medicine and Pharmacy “Carol Davila”, 020021 Bucharest, Romania; 2 Neonatology Department, Clinical Hospital of Obstetrics and Gynecology “Prof. Dr. P. Sârbu”, 060251 Bucharest, Romania; 3 Pediatric Hospital Ploiesti, 100326 Ploiesti, Romania; 4 Dermatology Department, “Elias” University Emergency Hospital, 011461 Bucharest, Romania; 5 Pediatrics Department, Doctor Victor Gomoiu’s Children Hospital, 022102 Bucharest, Romania; 6 Pediatrics Department, National Institute for Mother and Child Health “Alessandrescu-Rusescu”, 20382 Bucharest, Romania; 7 Neonatology Department, National Institute for Mother and Child Health “Alessandrescu-Rusescu”, 20382 Bucharest, Romania; 8 Marie Skłodowska Curie Children’s Emergency Hospital, 077120 Bucharest, Romania; 9 Emergency Hospital for Children “Grigore Alexandrescu”, 011743 Bucharest, Romania

**Keywords:** infantile hemangioma, propranolol, treatment

## Abstract

**Background/Objectives**: Infantile hemangioma (IH) is a common vascular tumor in neonates, influenced by multiple prenatal and perinatal factors. This study aimed to identify risk factors in both infants and mothers, assess their link to clinical characteristics and severity, and evaluate treatment outcomes when systemic propranolol therapy was administered. **Methods**: We conducted a retrospective observational study analyzing 43 infants under 12 months, including 11 neonates (<28 days) diagnosed with IH. Maternal and neonatal factors, diagnostic timelines, clinical presentation, and treatment efficacy were examined. Data analysis included descriptive statistics, focusing on gestational age, birth weight, Apgar scores, and the Infantile Hemangioma Referral Score (IHReS). **Results**: The study found a female predominance and a correlation between IH and pre-term birth (50%) and low birth weight (<2760 g, 51.16%). Maternal anemia (23%) and gestational hypertension (9%) were present in the cohort, but no statistical association with IH severity was found. A significant number (44.18%) were diagnosed within the first two weeks postpartum. The IHReS was inversely correlated with Apgar scores, with newborns scoring above 8 having a lower IHReS. Treatment with propranolol (1–3 mg/kg/day) was highly effective, resulting in significant lesion regression in most patients. Mild complications included sleep disturbances (12%) and diarrhea (9%). The most affected areas were the face/eyelid (32.55%), limbs (18.6%), and anterior thorax. Additionally, 42% of cases had an IHReS above 4, with multiple hemangiomas increasing severity. **Conclusions:** IH was common in pre-term and low-birth-weight infants, whereas the maternal comorbidities observed in this small cohort did not show a definitive association, underscoring the need for controlled studies. Early diagnosis, risk stratification, and timely propranolol therapy are crucial in achieving favorable outcomes. Further research is needed to assess long-term effects and evaluate risks of treatment rebound.

## 1. Introduction

Infantile hemangioma (IH) affects roughly 4–5% of infants, and meta-analyses show markedly higher odds in female, pre-term, low-birth-weight, and multiple-gestation newborns [1]. IHs are benign vascular tumors in infancy, but they often necessitate medical intervention in severe or highly visible cases. Since 2008, propranolol, a non-selective beta-blocker has become the first-line therapy for IH due to its high efficacy, predictable safety profile, and manageable administration through oral dosing [2,3,4]. Numerous clinical studies have evaluated its efficacy in inducing the regression of IH, as well as its safety compared to historical alternatives, such as corticosteroids, which were once the mainstay of treatment [5,6,7,8,9].

Across multiple randomized and real-world studies, propranolol achieves ≈90% regression of IH with a better safety profile than corticosteroids and is more cost-effective than surgery, making it the current standard of care [3,6,7,9,10,11,12,13,14,15,16]. However, because propranolol therapy entails systemic β-blockade in very young infants, its safety profile must be evaluated with particular care as it can lead to adverse effects such as hypoglycemia, bradycardia, hypotension, and bronchospasm [2,17,18].

As the timely diagnosis and comprehensive management of IH have been shown to improve outcomes and reduce the physical and psychosocial burdens associated with IH, the available literature strongly supports early propranolol use as a first-line therapy, while ongoing research aims to address the remaining knowledge gaps related to rebound growth, long-term outcomes, and personalized treatment protocols [19,20]. The aim of the current study is to describe perinatal risk factors, lesion severity (IHReS), and short-term clinical response to oral propranolol in infants with IH treated at a single tertiary center. Given these established benefits, we examined how perinatal risk factors interacted with early IH severity and real-world propranolol response in our region.

## 2. Materials and Methods

We performed a retrospective observational study at the Paediatric Department of Ploiesti Hospital from March 2023 to October 2024. All neonates and infants up to 12 months of age who began oral propranolol therapy for hemangioma during this period were eligible. Patients were excluded if they had pre-existing respiratory or cardiac disease, were older than 12 months, or required primary surgical management. Thus, the study cohort comprised 43 pediatric patients.

Before treatment, every child underwent a detailed history, physical examination, and baseline cardiologic assessment that included echocardiography. Cardiovascular monitoring was performed solely as an additional safety and parental reassurance measure; it was not a prerequisite for treatment and did not alter management. Lesion severity was quantified with the Infantile Hemangioma Referral Score (IHReS) at baseline and at each follow-up visit. Effectiveness was determined at monthly visits by pediatric dermatologists through the visual assessment of serial photographs and physical examination—IHReS. The response was recorded dichotomously as favorable (the noticeable clinical regression of color and/or size) or no improvement.

The initial dose of propranolol was 1 mg/kg/day; electrocardiographic monitoring was performed one and two hours after the first dose. The dose was subsequently increased to 2 mg/kg/day and then to 3 mg/kg/day, with ECG checks after each escalation and a median treatment duration of 6 months. Follow-up throughout this period consisted of repeated cardiologic and dermatologic reviews and soft-tissue ultrasonography to document treatment response and safety.

The data were analyzed using IBM SPSS Statistics version 25 and illustrated using Microsoft Office Excel/Word 2013. Quantitative variables were tested for normal distribution using the Shapiro–Wilk test and were written as such: the ones with normal distribution as averages with standard deviation and the ones that did not have a normal distribution as medians with interquartile ranges (IQRs). Quantitative variables were tested between two independent groups using Mann–Whitney U tests. Fisher’s exact test was used to determine the non-random associations between categorical variables with the Bonferroni method used for correction. We conducted a post hoc sample size estimation using GPower (v3.1). Based on the observed difference in IH severity (IHReS > 4) between pre-term (37.5%) and term infants (26.3%), an effect size w = 0.236 was calculated. Assuming a two-sided chi-squared test with α = 0.05 and power = 0.80, the minimum required total sample size would be 171 participants (approximately 86 per group). Given the current study’s sample of 43 patients, the analysis was likely underpowered to detect small-to-moderate associations. This supports the need for larger, prospective studies to validate these findings.

## 3. Results

**Figure 1:**
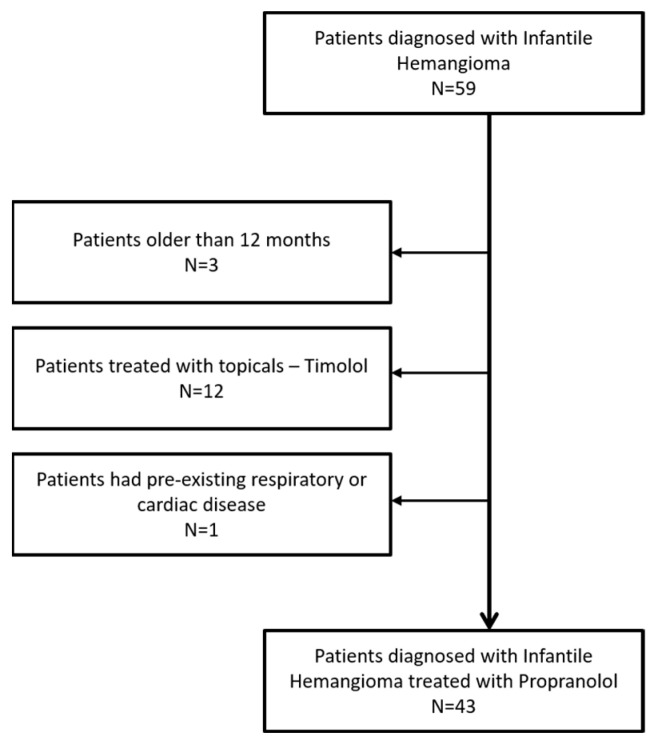
Cohort selection.

This study analyzed 43 neonates and infants under 12 months diagnosed with infantile hemangioma (IH) who were treated with propranolol (Figure 1). Sex distribution showed that 30 (69.8%) were female and 13 (30.2%) were male. There was no association between the sex of the patients and the distribution of the hemangiomas (*p* = 0.098) or the severity of the lesions (*p* = 0.860).

Most of the patients were pre-term 24 (55.8%), and 19 (44.2%) were full-term. Although the global test indicated a statistically significant association between prematurity and hemangioma localization (Fisher’s exact test, *p* = 0.040), post hoc comparisons of prematurity proportions across different localization groups did not reveal any significant differences after Bonferroni correction, as all categories were included in the same homogeneous subset. Hemangioma localization was classified into four anatomical regions: head and neck, trunk, upper limbs, and lower limbs (Table 1).

In terms of the IHReS, which assesses the severity of infantile hemangiomas and the need for intervention, 29/43 (67.4%) had a score ≤ 4 and 14/43 (32.6%) had an IHReS > 4. The IH developed with a clear predominance on the head and neck region and trunk, as is detailed in Table 1. A statistically significant association was observed between hemangioma localization on the head and neck and severity, as measured by the IHReS (Fisher’s exact test, *p* = 0.000; likelihood ratio, *p* = 0.000). However, post hoc comparisons between individual localization groups did not reveal statistically significant differences after Bonferroni correction, with all categories falling into the same homogeneous subset. There was also no association between the severity scores and multiple hemangiomas (*p* = 0.657).

**Figure 2:**
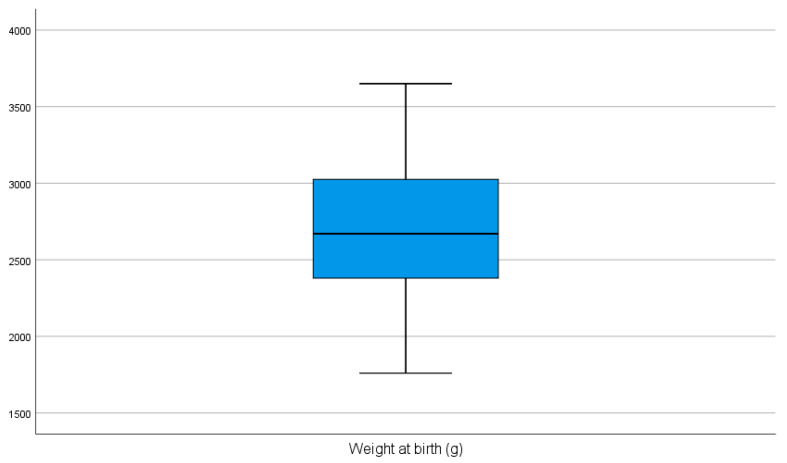
Weight at birth.

In this study the majority of subjects (32/43, 74.4%) had a normal birth weight (≥2500 g), while 10 subjects (23.3%) were classified as having a low birth weight (LBW, <2500 g), and only 1 subject (2.3%) had a very low birth weight (VLBW, <1500 g). No cases of extremely low birth weight (ELBW < 1000 g) were recorded. There was no association between the weight at birth and the distribution of the hemangiomas (*p* = 0.051). The weight at birth had a normal distribution (*p* = 0.82) and an average of 2714 ± 465 g (Figure 2).

**Figure 3:**
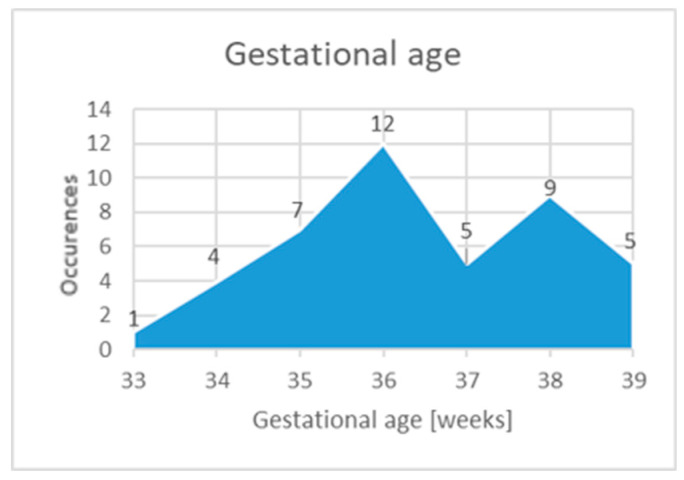
Gestational age.

The gestational age ranged from 33 to 39 weeks, with most newborns falling between 36 and 38 weeks. The gestational age at birth was not normally distributed (*p* = 0.022) and had a median of 36 (35–38) weeks (Figure 3). There was no association between the gestational age at birth and the severity of the lesions (*p* = 0.209).

Apgar scores, showed that 39 out of 43 subjects (90.7%) had an Apgar score ≥ 7, indicating a stable postnatal condition. However, four newborns (9.3%) had Apgar scores < 7, all of whom were in the LBW or VLBW categories (Table 2 and Table 3). The Apgar score at birth was not normally distributed (*p* < 0.001) and had a median of 8 (8-8) points. There was no association between the Apgar score at birth and the severity of the lesions (*p* = 0.387) (Table 2).

Most of the patients (58.1%) did not have any other conditions, while 13 (30.2%) had iron deficiency anemia, 3 (7%) had diaper rash, and 2 (4.7%) had other conditions. There was no association between the lack or presence of these conditions and the severity (*p* = 0.555) (Table 4).

**Figure 4:**
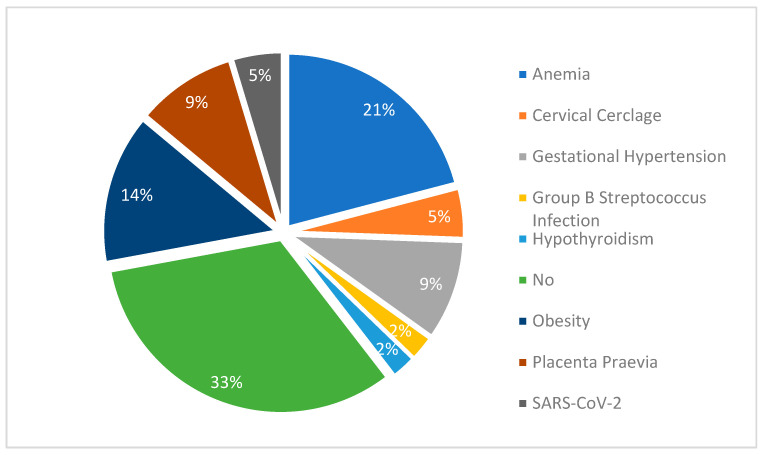
Maternal conditions.

Fourteen mothers (32.6%) had no chronic disease or pregnancy complication, while anemia affected 10 (23.3%), obesity affected 6 (14.0%), gestational hypertension affected 4 (9.3%), and placenta previa affected 4 (9.3%) (Figure 4). There was no association between maternal comorbidities and the severity of the hemangiomas (*p* = 0.748). Also, the majority of the mothers did not receive any medication during pregnancy (67.4%); 8 (18.6%) received iron supplements, 4 (9.3%) received methyldopa, 1 (2.3%) received antibiotics, and another 1 of them received levothyroxine. There was no association between any of those medications and the severity of the child’s lesions (*p* = 0.547 (Table 5).

All 43 infants showed a favorable response after dose escalation to 1–3 mg/kg/day. No cases of ‘no improvement’ or treatment failure were observed. Mild, self-limited adverse events occurred in 11/43 (25.6%) of patients (sleep disturbance, 5; diarrhea, 4; emesis, 2) (Table 6). The characteristics of the population can be found in Table 3.

**Figure 5:**
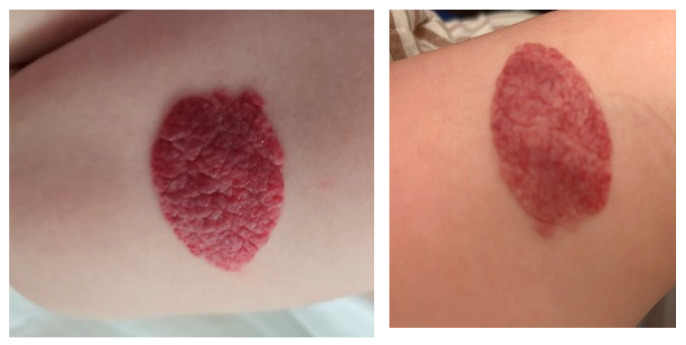
Lesion with significant improvement, flattened after 7 months of treatment.

**Figure 6:**
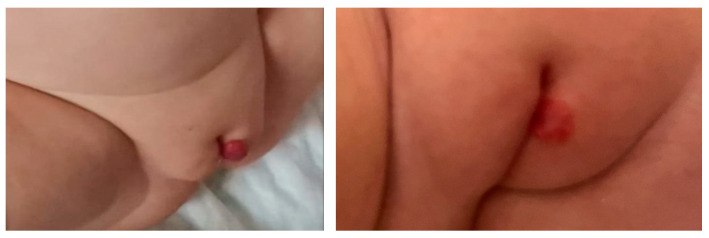
Vulvar lesion regression after 6 months of treatment.

**Figure 7:**
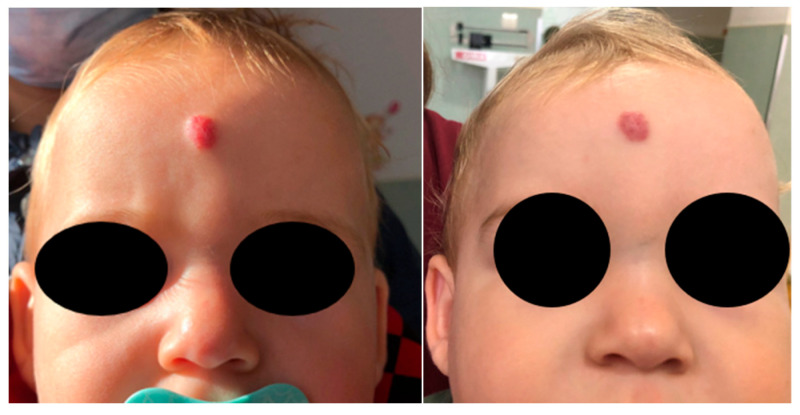
Frontal hemangioma regression after 2 months of treatment.

Some examples of the evolution of patients after treatment with propranolol can be found in Figure 5, Figure 6 and Figure 7.

## 4. Discussion

The specialized literature identifies that factors such as a low birth weight, female sex, placental abnormalities, maternal tobacco use during pregnancy, twin or multiple gestation, fertility treatment, progesterone use, and a family history of infantile hemangiomas (IHs) are associated with the development of IH [1,21].

Consistent with a meta-analysis by Hunjan et al., which examined 869 cases diagnosed with IH and reported a higher incidence in females, our study similarly noted a predominance of IH in females, accounting for 79% of cases, compared to 21% in males. Several prenatal conditions—gestational diabetes mellitus, pre-eclampsia, and gestational hypertension—have been reported in association with a higher incidence of IH, although the underlying causal mechanisms remain unclear [22]. Additionally, it was observed in our analysis that in nine cases, the mothers had anemia during pregnancy, four were diagnosed with pregnancy-induced hypertension, four had placenta previa abnormalities, and one isolated case was diagnosed with hypothyroidism during pregnancy. This singular case aligns with the findings of Igarashi et al., and although the evidence is based on only a few reports, it raises the possibility that maternal thyroid dysfunction may contribute to IH pathogenesis—a hypothesis that warrants investigation in larger, controlled studies [23]. The high prevalence of maternal anemia observed in our cohort may be explained by the proposed pathophysiology of IH, in which local hypoxia plays a key role. Hypoxic conditions are known to activate the hypoxia-inducible factor (HIF) pathway, which has been implicated in the development of IH. Maternal anemia may contribute to a hypoxic intrauterine environment, thereby increasing the likelihood of hemangioma formation at birth [24].

In terms of gestational age, our findings indicate that prematurity considerably influences the mechanisms underlying IH in neonates (Figure 6). Over 50% of the cases involved pre-term infants, specifically between 34 and 37 gestational weeks. Notably, a substantial proportion—approximately 17 cases (39.5%)—was classified as late pre-term. While the overall association between prematurity and hemangioma localization was statistically significant, pairwise comparisons did not reveal significant differences between individual localization sites, possibly due to the small sample. The literature also indicates a higher incidence of IH among premature infants, with Goelz et al. showing in their review of IH therapy in premature newborns that the occurrence of hemangiomas escalates as gestational age decreases [25].

Regarding birth weight, most infants in our cohort fell within the normal range, and we found no significant association with hemangioma severity. Furthermore, the Apgar score at birth was observed to be directly proportional to gestational age. Thus, among the study population, 33 cases (74.74%) had Apgar scores of 7 and 8. A correlation was also recorded between the Apgar score and the IHReS, with the two being inversely proportional. Thus, newborns with an Apgar score greater than 8 exhibited significantly lower IHReS scores. In contrast, cases that received an Apgar score between 7 and 8 accounted for a percentage of 76.74% of the patients, all of whom had an IHReS score exceeding 4 points. This finding highlights the increased likelihood of a low Apgar score manifesting clinically as a risk factor for IH. It has also been shown that multiple gestation is correlated with an increased incidence of IH [1]. However, in our analysis, only two cases were observed in infants from twin pregnancies.

Regarding the diagnostic timeline, in our cohort, we had a higher incidence of diagnoses occurring within the first eight weeks postpartum. Specifically, 19 cases (44.18%) were identified in the first two weeks, and 8 cases (18.6%) were identified in week four, with the remaining patients being diagnosed after the eighth week of life.

According to the location of the hemangioma, the infantile Hemangioma Referral Score (IHReS) was implemented, focusing on the anatomical regions affected by infantile hemangioma, including the face, neck, limbs, thorax, abdomen, and genital area [26]. The location, depth, and stage of advancement, along with the severity of the lesion, helped in predicting the response to treatment and the patient’s prognosis [27]. In our study, 14 cases (32.55%) involved IH on the eyelid or facial area, 8 cases (18.6%) affected the upper or lower limbs, 5 cases were located on the anterior thorax, and a single case was found involving the lumbosacral region. In addition, some cases comprised more than five hemangiomas distributed across different body sites, which represented a criterion for a higher IHReS. The severity of the diagnosis is determined by the IHReS values, with a score exceeding 4 points announcing a reserved prognosis and the potential for complications [28]. While the overall association between hemangioma localization and severity (IHReS) was statistically significant, pairwise comparisons between localization sites did not demonstrate significant differences, which may be attributed to the small sample size.

An important consideration is the association of certain comorbidities or the classification of IH within a syndrome. In our study, approximately 58.13% of the cases presented without underlying conditions, while 27.9% were associated with iron deficiency anemia and three cases exhibited diaper rash. De Ravin et al. published a multicenter, randomized clinical trial emphasizing the management of IH through specific scoring systems. These scores evaluated factors such as the degree of functional impairment such as visual impairment, the involvement of internal organs, and associated structural abnormalities included in PHACE syndrome [29]. Patients presenting with hemangiomas larger than 5 cm or multiple hemangiomas were managed by a multidisciplinary team comprising pediatricians, a pediatric cardiologist, an ophthalmologist, a dermatologist, and a pediatric surgeon in order to determine the appropriate management.

Early diagnosis and appropriate risk stratification using IHReS enabled the identification of cases that could benefit from beta-blocker therapy. In our cohort, systemic treatment with propranolol was effective in all patients recruited. During the inclusion period, only one case diagnosed with atrial septal defect had a contraindication to beta-blocker administration, and an isolated case received topical treatment with Timolol^®^ instead. Treatment was administered over a period of 6 months, resulting in favorable outcomes, particularly evident in the regression of the hemangioma. Complications associated with the therapy included mild sleep disorders (12%), diarrhea (9%), and vomiting (5%). It should be noted that 74% experienced no complications throughout the treatment period. These results align with the literature, which has found that systemic treatment with propranolol is safe and effective, but in most cases, just as in our analysis, mild side effects such as diarrhea and vomiting can be observed [30,31]. Given that beta-blockers primarily target cardiac function, the safety profile of propranolol has been extensively studied. Cardiovascular symptomatic adverse effects are particularly rare and generally resolved upon treatment discontinuation [32]. Accordingly, the baseline ECG and brief post-dose monitoring in our protocol served purely as supportive measures to reassure caregivers; they did not influence eligibility or dosing, and centers with limited resources or extensive experience may reasonably omit them. More severe or life-threatening occurrences, such as symptomatic hypoglycemia, severe respiratory disorders, and significant sleep disturbances, have been reported [33]. However, adverse events are generally mild and manageable within standardized treatment protocols [3,18,34]. Current guidance permits propranolol to be initiated safely in otherwise healthy infants in an outpatient setting, although some centers opt for brief in-clinic observation at the first dose according to local protocols and caregiver preference. However, in high-risk groups, such as those with PHACE syndrome, propranolol use requires careful adaptation to avoid the exacerbation of cerebrovascular anomalies [2,18,34].

Despite propranolol’s success, some questions remain. Rebound growth after the cessation of therapy occurs in 4–21% of cases, necessitating the reinitiation of treatment in select patients [2,35,36]. Recurrence rates for our cohort could not be assessed because systematic post-therapy surveillance was not performed, and this limitation should be considered when interpreting our findings. While early initiation (within the first 5–6 months of life) yields the most favorable outcomes, further research is needed to define optimal treatment windows and determine patient-specific predictors of efficacy or rebound risk [4,37,38].

This study has several limitations. First, the sample size was relatively small, and the analysis was underpowered to detect small-to-moderate associations. Also, because the study lacked a control group, the uncontrolled design precluded any inference about maternal and fetal risk factors; larger, controlled studies are required to clarify these associations. In addition, effectiveness was based solely on visual assessment without objective volumetric imaging; while this reflects common real-world practice, future studies should incorporate standardized imaging to quantify regression. Taken together, our data reinforce early outpatient propranolol initiation and provide local benchmarks for IHReS-guided referral.

## 5. Conclusions

In conclusion, this study highlights the multifactorial nature of infantile hemangioma (IH) and underscores the importance of early diagnosis, risk stratification, and individualized management. Consistent with the previous literature, we observed more IH in females, pre-term infants and those with a low birth weight. Maternal anemia, gestational hypertension, and hypothyroidism were present in a subset of mothers but showed no statistical association with lesion severity, and our uncontrolled design precluded us from inferring their role in IH risk. The IHReS proved useful in stratifying disease severity throughout the treatment period; propranolol remained effective and well tolerated, supporting its first-line status. Nevertheless, several methodological limitations temper these conclusions: the cohort was modest and—most importantly—no control group was available for direct comparison. These constraints may limit the statistical power and generalizability, and they preclude firm causal inferences. In the future, larger, controlled studies with extended follow-up are required to validate our associations, refine the predictive value of the risk factors that we identified, and clarify long-term outcomes.

## Figures and Tables

**Table 1:** Anatomical distribution of IH.

Location	Number of Subjects
Upper Limb	6
- Right arm and forearm	1
- Right forearm	1
- Left arm	2
- Upper limb (unspecified)	1
- Right upper limb (part of multiple hemangiomas)	1
Lower Limb	5
- Left ankle	1
- Left leg	2
- Left thigh	1
- Lower limb (unspecified)	1
Trunk	13
- Abdominal wall	2
- Anterior chest	4
- Posterior chest	1
- Lumbosacral area	1
- Labial and left lumbar area	1
- Labial and right hemithorax	1
- Right retroauricular area and right hemithorax, perimammary	1
- Scalp and intergluteal area	1
- H. multiple anterior/posterior chest, right upper limb	1
Head and neck	19
- Facies	11
- Eyelid	3
- Lower lip	2
- Submandibular	1
- Scalp	4

**Table 2:** Apgar score and IHReS Severity Score.

Apgar Score	≤4	>4	Fisher’s Exact Test
7	6 (60%)	4 (40%)	*p* = 0.387
8	15 (65.2%)	8 (34.8%)	
9	3 (37.5%)	5 (62.5%)	
10	1 (50%)	1 (50%)	

**Table 3:** Neonatal birth weight classification, IHReS, and Apgar score (5 min) distribution.

Birth Weight Category	Sex Distribution (M/F)	Gestational Age (Range in Weeks)	IHReS ≤ 4 (Count)	IHReS > 4 (Count)	Apgar Score < 7 (Count)	Apgar Score ≥ 7 (Count)
Normal Birth Weight (≥2500 g)	8 M/24 F	34–39 weeks	21	11	0	32
Low Birth Weight (LBW < 2500 g)	5 M/5 F	33–38 weeks	8	2	3	7
Very Low Birth Weight (VLBW < 1500 g)	0 M/1 F	33 weeks	0	1	1	0
Total Subjects	13 M/30 F	33–39 weeks	29	14	4	39

**Table 4:** Child comorbidities and IHReS Severity Score.

Child’s Comorbidities	≤4	>4	Fisher’s Exact Test
Diaper Rash	3 (100.0%)	0 (0%)	*p* = 0.555
Iron Deficiency Anemia	7 (53.8%)	6 (46.2%)	
Other	1 (50%)	1 (50%)	
No Comorbidities	25 (58.1%)	18 (41.9%)	

**Table 5:** Mother’s medication during pregnancy and IHReS Severity Score.

Mother’s Medication During Pregnancy	≤4	>4	Fisher’s Exact Test
Antibiotics	1 (100.0%)	0 (0%)	*p* = 0.547
Iron supplements	6 (75%)	2 (25%)	
Methyldopa	2 (50%)	2 (50%)	
Levothyroxine	0 (0%)	1 (100.0%)	
No medication	16 (55.2%)	13 (44.8%)	

**Table 6:** Complications during treatment with propranolol.

Complications	Number (%)
No complications	32 (74.4%)
Sleep disturbance	5 (9.3%)
Diarrhea	4 (4.7%)
Emesis	2 (4.7%)

## Data Availability

Data is contained within the article.

## Abbreviations

The following abbreviations are used in this manuscript:

IH	Infantile hemangioma
IHReS	Infantile Hemangioma Referral Score
